# RETRACTION: **Fibronectin Promotes Migration and Invasion of Ovarian Cancer Cells through Up‐Regulation of FAK–PI3K/Akt Pathway**

**DOI:** 10.1002/cbin.70190

**Published:** 2026-07-29

## Abstract

**RETRACTION:** Nasser Ghaly Yousif, “Fibronectin Promotes Migration and Invasion of Ovarian Cancer Cells through Up‐Regulation of FAK–PI3K/Akt Pathway,” *Cell Biology International* 38, no. 1 (2014): 85–91, https://doi.org/10.1002/cbin.10184.

The above article, published online on 21 September 2013 in Wiley Online Library (wileyonlinelibrary.com), has been retracted by agreement between the journal Editor‐in‐Chief, Xuebiao Yao; the International Federation for Cell Biology; and John Wiley & Sons Ltd. The retraction has been agreed upon following concerns raised by a third party. An investigation confirmed several duplications of western blot bands within figures 2A, 2B, and 3A. Further duplications of western blot bands were found between figures 2A and 4A. In addition, large portions of the introduction were taken from an earlier article published by a different author group, without attribution. Although the author initially responded to the concerns and agreed to provide the original data, they failed to provide any supporting information or to respond to subsequent reminders. They also did not provide a plausible explanation for the reused text in the introduction. The editors consider the results and conclusions of this article to be compromised. The author did not respond to our notice of retraction.

**RETRACTION:** Nasser Ghaly Yousif, “Fibronectin Promotes Migration and Invasion of Ovarian Cancer Cells through Up‐Regulation of FAK–PI3K/Akt Pathway,” *Cell Biology International* 38, no. 1 (2014): 85–91, https://doi.org/10.1002/cbin.10184.

The above article, published online on 21 September 2013 in Wiley Online Library (wileyonlinelibrary.com), has been retracted by agreement between the journal Editor‐in‐Chief, Xuebiao Yao; the International Federation for Cell Biology; and John Wiley & Sons Ltd. The retraction has been agreed upon following concerns raised by a third party. An investigation confirmed several duplications of western blot bands within figures 2A, 2B, and 3A. Further duplications of western blot bands were found between figures 2A and 4A. In addition, large portions of the introduction were taken from an earlier article published by a different author group, without attribution. Although the author initially responded to the concerns and agreed to provide the original data, they failed to provide any supporting information or to respond to subsequent reminders. They also did not provide a plausible explanation for the reused text in the introduction. The editors consider the results and conclusions of this article to be compromised. The author did not respond to our notice of retraction.

